# Use of Imaging to Prevent Unnecessary Workup and Answer a Clinical Question

**DOI:** 10.7759/cureus.87158

**Published:** 2025-07-02

**Authors:** Petar Martinovski, Morgan Sly, David McVinnie, Peter Joseph Massa

**Affiliations:** 1 Interventional Radiology, Henry Ford Health, Detroit, USA

**Keywords:** academic radiology, interventional radiologist, interventional radiology, minimally invasive interventional radiology, radiology teaching

## Abstract

With a fair percentage of all medical care deemed unnecessary for diagnosis and treatment, it is paramount that clinicians consider the rationale for each order and whether it is truly warranted. We present the case of a 71-year-old male with a history of Roux-en-Y gastric bypass who presented with choledocholithiasis and was found to have an incidental finding of a persistently narrowed segment of the right bile duct. With the usage of multiple imaging modalities, particularly CT angiography and intravascular ultrasound via an endobiliary route during biliary drain management, it was confirmed that the narrowing of concern was caused by a benign vascular compression by the right hepatic artery as opposed to a malignancy. This case demonstrates how minimally invasive imaging can reduce further potentially unnecessary tests, and thus costs, for patients.

## Introduction

With about 20% of medical care overall not being necessary, including 22% of prescriptions, 25% of tests, and 11% of procedures, it is crucial for clinicians to ensure the rationale for each is absolutely necessary [1]. As mentioned in the Choosing Wisely Campaign, this is crucial to not only reduce patient costs but also any unnecessary burdens on patients’ well-being due to potential diagnoses that turn out to be inaccurate [2]. In this case, we will see an example of how minimally invasive imaging performed during treatment for the primary condition prevented future workup and costs for the patient and determined that their bile duct narrowing was due to a benign vascular compression.

## Case presentation

**Figure 1:**
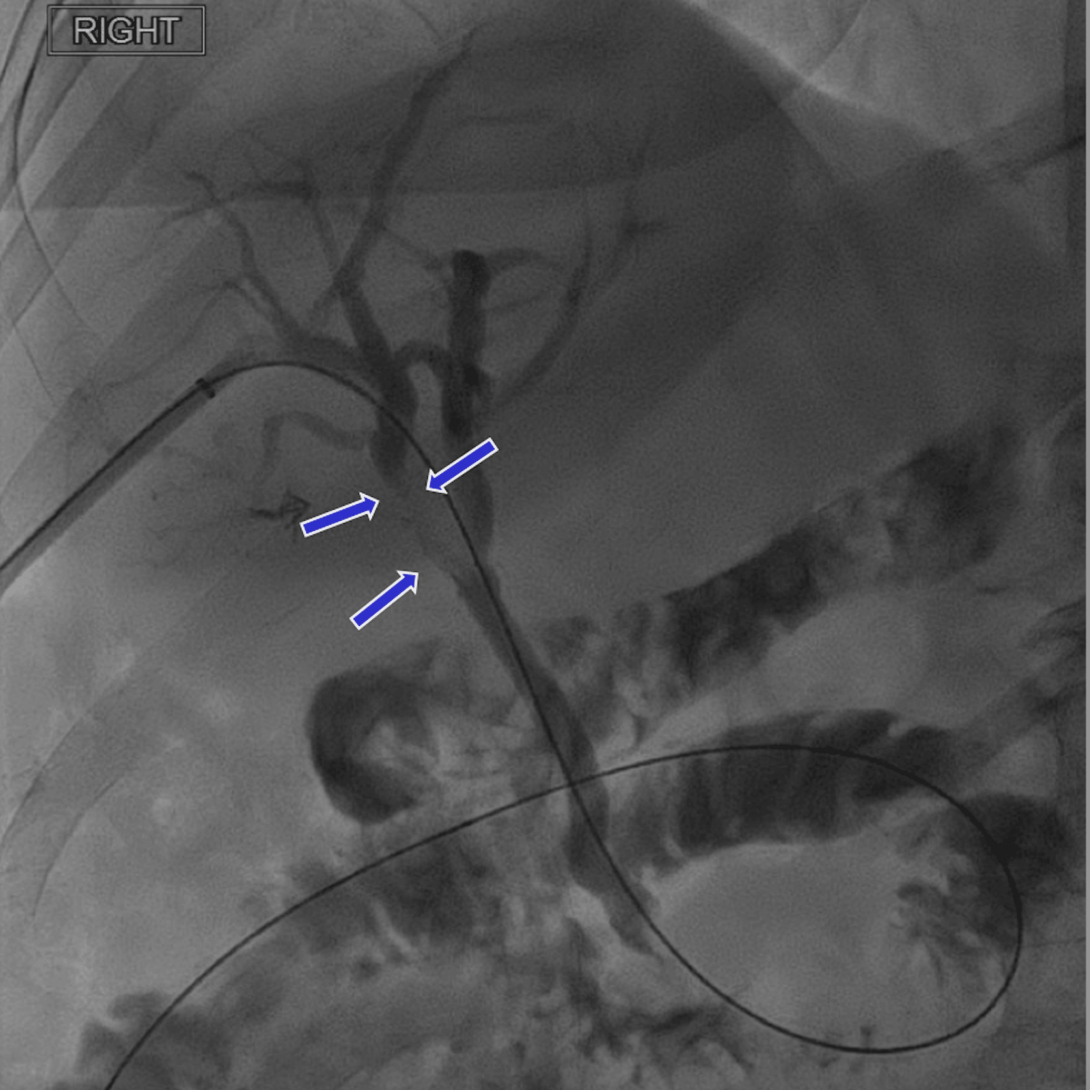
Blue arrows point to the persistent narrowing of the right hepatic bile duct.

**Figure 2:**
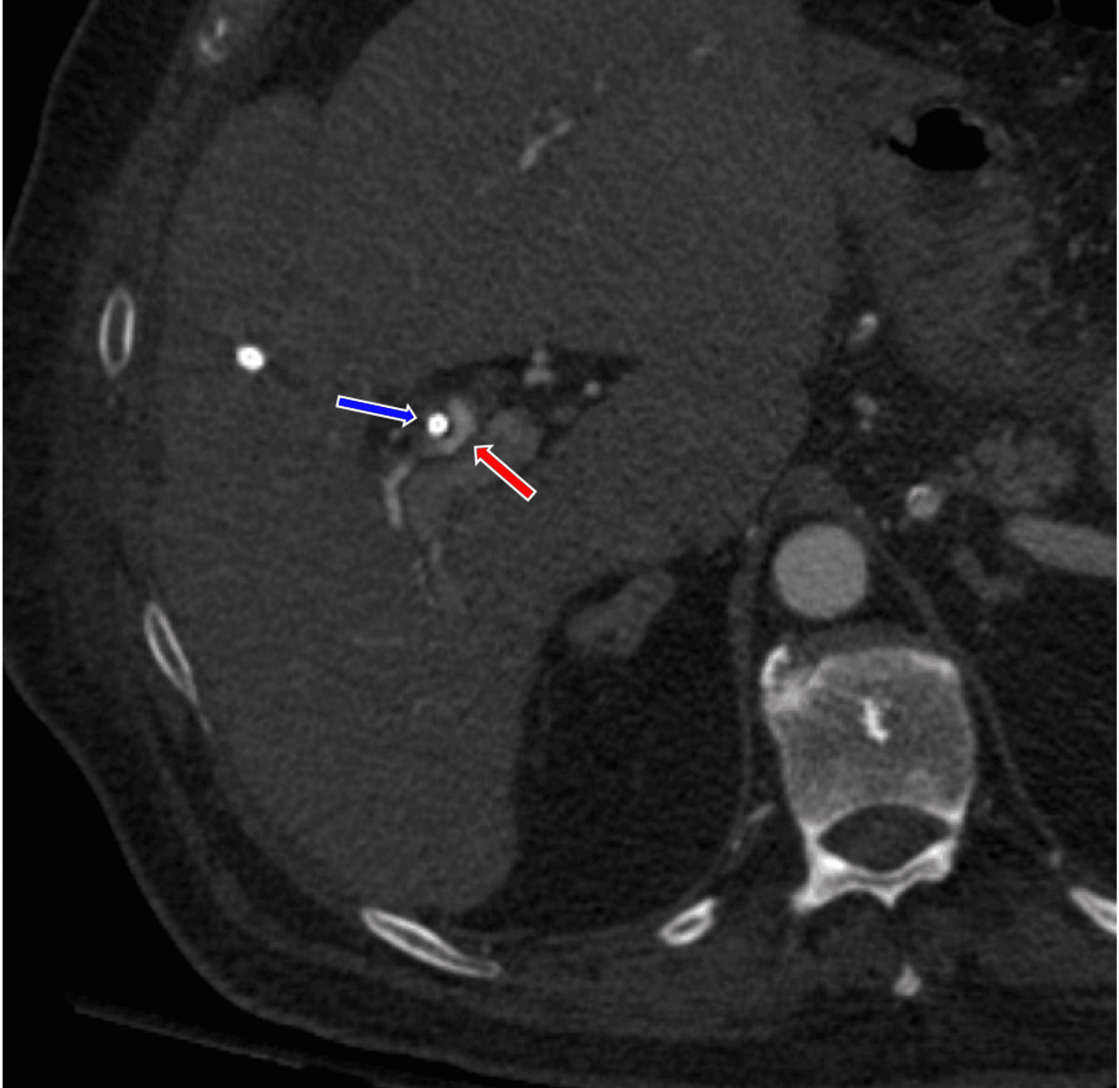
The blue arrow shows the biliary drain passing through the right hepatic bile duct, and the red arrow shows the adjacent right hepatic artery.

**Figure 3:**
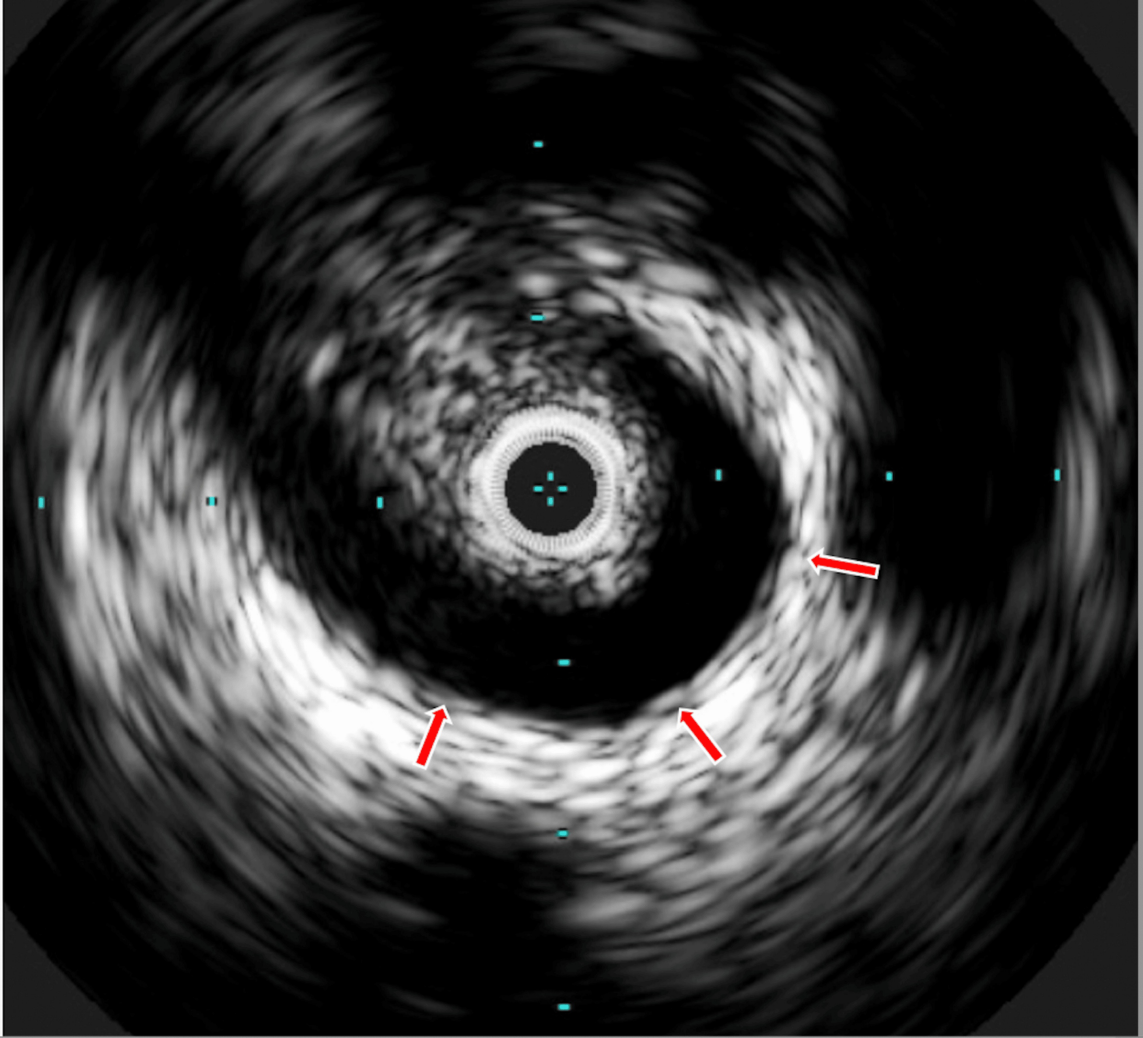
Intravascular ultrasound from inside the right hepatic duct at the level of the narrowing shows the immediately adjacent right hepatic artery (red arrow).

A 71-year-old male with a prior history of Roux-en-Y gastric bypass presented with symptoms of jaundice. He was diagnosed with choledocholithiasis, which was managed with a percutaneous biliary drain, as he was not amenable to endoscopic retrograde cholangiopancreatography due to his previous bypass surgery. Initial cholangiograms showed a small amount of debris, sludge, or small stones in the distal common bile duct, which was cleared with a Fogarty balloon. Additionally, during his admission, he had a complication of ascending cholangitis that was treated with a proper antibiotic regimen. Despite appropriate management, subsequent cholangiograms showed a segment of the right bile duct that was persistently narrowed (Figure 1). Potential causes could be from cancer, inflammation, or congenital variation, among others. Close examination of CT of the pancreas with CT angiography done during the patient’s initial workup showed the right hepatic artery running close to the right bile duct (Figure 2), suggesting that the bile duct narrowing could be from a benign vascular compression. To test this theory, intravascular ultrasound performed during removal of the drain confirmed that the right bile duct was being compressed by the right hepatic artery (Figure 3). The duct was demonstrated to be functionally patent, and no further procedures were indicated. Since his discharge, he has not had any complications or new occurrences.

## Discussion

The incidental compression described previously from the right hepatic artery on the right bile duct appears to describe a case of right hepatic artery syndrome. This occurs when the right hepatic artery compresses the extrahepatic bile duct, with complications including cholangitis, obstructive jaundice, among others. On our literature search, we found a description of a case of right hepatic artery syndrome that was also complicated by Mirizzi syndrome, a rare cause of biliary obstruction where the swelling of gallstones that are stuck in the cystic duct causes a blockage in bile flow. There are also a few variants of this particular anatomical rarity found in the literature. One such example involves an arterial ringed compression of the proper hepatic artery around the common bile duct. Another is compression of the right hepatic artery of the proximal common bile duct [3-6]. From our search, this appears to be only one of a small number of cases we can find on compression of the right hepatic artery on the right bile duct.

Looking closely at our case, it is possible that our patient’s choledocholithiasis and, by extension, his complication of ascending cholangitis might in some ways have been contributed to by this unique anatomical variation. However, this is less plausible given that his previous history of Roux-en-Y gastric bypass by itself has a risk of predisposing one to the stone formation [7-9]. Additionally, as the stones were found in the distal common bile duct, this also lowers this possibility. While we cannot know for certain, given that in the three years since his symptoms resolved, he has had no reoccurrence, and yet the benign compression is still present, this lowers the probability of its contribution to his presenting symptoms.

## Conclusions

Even though the predisposing cause of the choledocholithiasis is most likely not due to the benign compression described, this case shows the benefit of using minimally invasive imaging modalities to answer a critical clinical question. The imaging modalities used were either done as part of the original workup or, as in the case of the intravascular ultrasound, done during the biliary drain removal; hence, no additional cost or procedure time was incurred by the patient. The resolution of the question of what caused the bile duct narrowing prevented additional workup, which avoided unnecessary discussions about potentially serious topics such as cancer, directly benefiting patient care.
